# Nivolumab-Induced Encephalitis in Hereditary Leiomyomatosis and Renal Cell Cancer Syndrome

**DOI:** 10.1155/2018/4273231

**Published:** 2018-01-17

**Authors:** Benjamin Chaucer, Abriella Stone, Augustus Demanes, Shawn M. Seibert

**Affiliations:** ^1^University of Illinois College of Medicine at Peoria, Peoria, IL, USA; ^2^Illinois Cancer Care, Peoria, IL, USA

## Abstract

The treatment of cancer is a rapidly evolving field. As more chemotherapeutic agents become available, reporting the side effects of these agents in clinical practice becomes increasingly important. Nivolumab is one of the chemotherapeutic agents commonly used for treatment of renal cell carcinoma, metastatic melanoma, and metastatic non-small cell lung cancer. While common side effects are known and well documented, encephalitis is documented as an extremely rare side effect. We present the case of an extremely rare side effect to a common chemotherapeutic agent.

## 1. Introduction

Nivolumab, marketed as Opdivo, is a human monoclonal antibody used in the treatment of advanced renal cell carcinoma. Common side effects include cough, nausea, asthenic conditions, rash, and dyspnea. An extremely rare complication seen in 0.2% of patients taking nivolumab is immune-mediated encephalitis [1]. Nivolumab is a relatively new pharmaceutical agent, and side effects are still being discovered in clinical practice. Nivolumab may have long reaching effects on patients. As many as 44% of patients on nivolumab experience a drug delay for an adverse reaction [1]. In patients with advanced renal cell carcinoma, nivolumab is a common therapy. Reed's syndrome is an exceedingly rare genetic disorder. Effected patients often experience renal cell carcinoma and leiomyomas due to an inherited mutation in the fumarate hydratase (FH) gene. To date, only 100 families worldwide have been diagnosed with the mutation [2]. Given the severity of this cancer, aggressive therapy is often required. We present the case of an exceedingly rare familial cancer syndrome treated with nivolumab resulting in encephalitis.

## 2. Case Report

Our patient is a 44-year-old male who was brought in by ambulance for altered mental status. As per the patient's wife, the week prior to admission he began staring off in space and responding to internal stimuli. His mentation deteriorated, and he began hallucinating and became aggressive, at which point she brought him to the emergency department. Six months prior to admission, he was diagnosed with metastatic renal cell carcinoma and underwent primary cancer removal with subsequent right nephrectomy. He was found to have multiple leiomyomas and was referred to the National Cancer Institute in Maryland. Genetic testing showed autosomal dominant Hereditary Leiomyomatosis and Renal Cell Carcinoma (HLRCC). Follow-up L-spine MRI for back pain showed extensive metastasis to the lumbar spine, sacrum, and right and left ilium. He was started on targeted immunotherapy of nivolumab and given a single dose of 240 mg two weeks prior to admission. On presentation, he was febrile and unable to communicate. He was acutely encephalopathic and responding to internal stimuli. During the interview, he was experiencing and responding to auditory, visual, and tactile hallucinations. On admission, his blood pressure was 122/86 with a pulse of 118 and temperature of 100.8°F. Chest X-ray showed no signs of pneumonia or metastases. Brain MRI showed no evidence of metastases or lymphoreticular disorder. Blood work showed an elevated white blood cell count of 16.61, unchanged from a previous admission due to home medication of Decadron. Serum Na was 132 with AST/ALT 36/72, TBili 0.5, alkaline phosphatase 246, and a creatinine of 2.0 at his baseline. Urinalysis performed in the ED showed packed hyaline casts without superimposed urinary tract infection. Blood cultures were drawn, and he was started on normal saline and oxygen. His home medications included methadone, naproxen, omeprazole, oxycodone, risperidone, hydromorphone, trazodone, benzonate, dexamethasone, buproprion, cyclobenzaprine, and ondansetron. Given the nature of the patient's pain and the severity of his illness, all of his home medications were continued. He was started on Haldol for agitation and transferred to the medical intensive care unit for treatment of encephalopathy. On examination, he exhibited garbled speech and continued encephalopathy. Initial attempts to sedate the patient using ketamine were effective and resulted in increased lucidity. He eventually became hypertensive and required treatment with Precedex while in the MICU. After 5 days in the MICU, the patient was found to be speaking in short sentences and was alert, awake, and oriented to person, place, and time with no signs of encephalopathy. Blood cultures throughout the stay showed no growth. Given the patient's lack of metabolic abnormalities, acute infection, continuation of all home medication throughout the hospital stay, and temporal resolution of symptoms after removal of nivolumab, he was diagnosed with nivolumab-induced encephalitis. The patient was discharged home on his home medications and scheduled for outpatient radiation therapy with discontinuation of nivolumab. After a short stay at home, patient was advanced to hospice and soon after passed away from metastatic renal cell carcinoma.

## 3. Discussion

Reed's syndrome, also known as hereditary leiomyomatosis and renal cell cancer (HLRCC) syndrome, is an autosomal dominant familial syndrome, first described in 1973. Cutaneous leiomyomas and uterine fibroids are the most prevalent features of this syndrome, with some patients experiencing renal cell carcinoma. Renal cell carcinoma, when present, is often solitary and unilateral and tends to be aggressive with early metastasis [3]. Treatment of HLRCC is often a two step approach using surgical removal of solid tumors and then treatment with targeted immunotherapy. HLRCC is characterized by a mutation in the fumarate hydratase (FH) gene. The product of this gene is utilized in the Krebs cycle. While the mutated gene in HLRCC is understood, its effect on the patient is poorly defined. The currently accepted hypothesis is that it results in increased dependence on glycolysis and therefore results in a pseudohypoxia [4]. There is evidence that the mutation causes an increase in oxidative damage. Cell lines with this mutation have an increase in the generation of reactive oxygen species and increased expression of hypoxia-inducible factor 1 alpha which is necessary for the development of pseudohypoxia. Nivolumab is an anti-PD-1 receptor antibody. PD-1 is a programmed cell death protein expressed on the surface of B- and T-cells. Many tumors express PD-L, which activates the PD-1 protein and begins a cascade that results in apoptosis. The end product is the suppression of the host immune systems' ability to fight the tumor. By blocking this interaction, nivolumab serves to increase the host immune response against the tumor. It is used in the treatment for a variety of tumor types including advanced renal cell carcinoma. It is often used in tandem with ipilimumab, an anti-CTLA-4 antibody to further boost immune function [5]. Common adverse reactions include fatigue, rash, GI, and respiratory symptoms. Adverse neurological effects represent an exceedingly rare complication of nivolumab [1]. More common side effects include tremor, cerebral edema, multifocal CNS demyelination, optic neuritis, restless leg syndrome, impaired memory, and hypersomnia [6]. Neurological side effects account for 1–3% of all adverse reactions due to nivolumab with immune-related encephalitis only seen in 0.2% of patients on nivolumab [6, 7]. In our patient, the last administered dose of nivolumab was given 2 weeks prior to his initial change in mental status. This delay in adverse reaction is well documented in nivolumab and may be present in as many as 44% of patients receiving treatment. In our patient, as the time to exposure increased we saw a return to baseline. Our patient continued all prior to admission medications and was found to have no metabolic abnormalities or acute infections and was therefore diagnosed with nivolumab induced-encephalitis. In some cases, a more aggressive approach may be required. There is no currently defined connection between a mutation in the FH gene and the metabolism of nivolumab. Given the lack of research, we do not know if this mutation played a role in the development of our patients' encephalopathy. Compounding the difficulty in acquiring data is the fact that not all patients with HLRCC develop renal cell cancers and may therefore never be treated with targeted immunotherapy. This case report highlights the need for further research into nivolumab as well as rare genetics disorders such as HLRCC.
